# Socioeconomic deprivation is associated with worse in-hospital survival after isolated coronary artery bypass grafting in the UK

**DOI:** 10.1093/icvts/ivaf119

**Published:** 2025-05-21

**Authors:** Jeremy Chan, Pradeep Narayan, Jacie Jiaqi Law, Tim Dong, Gianni D Angelini

**Affiliations:** Bristol Heart Institute, University of Bristol, Bristol, UK; Rabindranath Tagore International Institute of Cardiac Sciences, Narayana Health, Kolkata, West Bengal, India; Bristol Heart Institute, University of Bristol, Bristol, UK; Bristol Heart Institute, University of Bristol, Bristol, UK; Bristol Heart Institute, University of Bristol, Bristol, UK

**Keywords:** coronary artery bypass grafting, socioeconomic deprivation, index of multiple deprivation

## Abstract

**OBJECTIVES:**

Previous studies have identified a correlation between socioeconomic deprivation and poorer outcomes following cardiac surgery in the USA, where healthcare is predominantly delivered through private system. However, the influence of socioeconomic deprivation in countries with universal healthcare systems, such as the UK, has been less extensively investigated. Therefore, we used the index of multiple deprivation (IMD) to evaluate the impact of socioeconomic status on early clinical outcomes following coronary artery bypass grafting (CABG) in the UK.

**METHODS:**

All patients who underwent elective/urgent isolated CABG between 2008 and 2019 in the UK were included. The IMD, along with other perioperative characteristics, were incorporated into a regression model to determine factors associated with in-hospital mortality.

**RESULTS:**

The analysis included 182 911 patients (median age: 67.3 years, 82.13% male). Patients were categorized into five groups based on IMD, 1: most deprived to 5 the least: 1 = 30 564, 2 = 30 815, 3 = 59 161, 4 = 31 891 and 5 = 30 480. Patients from the most socioeconomically deprived areas exhibited markedly higher rates of comorbidities and risk factors such as diabetes and had a higher rate of urgent surgical intervention. There is a small increase in in-hospital mortality when socioeconomic status declined, with rates of 1.30, 1.30, 1.24, 1.14 and 1.15% for group 1–5, respectively. Socioeconomic deprivation, particularly in income and education, was associated with an increase in in-hospital survival.

**CONCLUSIONS:**

Socioeconomic deprivation, particularly in income and education, is associated with higher burdens of comorbidity and a small decrease in-hospital survival after CABG in the UK. This suggests that these factors may play a critical role in clinical outcomes even in a universal healthcare system.

## GRAPHICAL ABSTRACT

**Figure:**
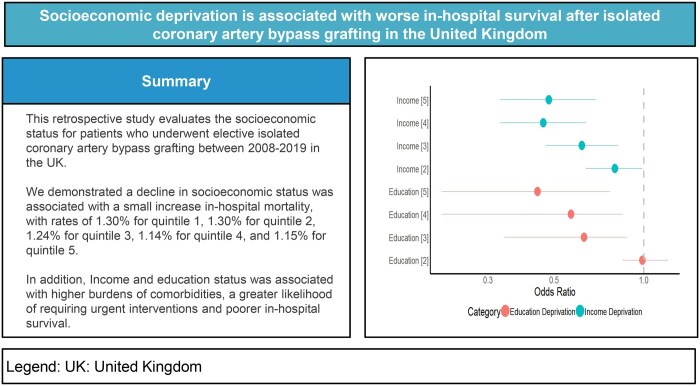

## INTRODUCTION

Socioeconomic deprivation, also known as socioeconomic distress, has been reported to increase the incidence of perioperative complications, short- and long-term mortality, readmission rate and failure to rescue in cardiac surgery [1–7]. However, there is no universally agreed definition of socioeconomic deprivation, and the specific components included can vary across nations [8]. Existing research also employs diverse definitions when reporting their findings. In addition, there are few contemporary data examining the implication of socioeconomic deprivation on coronary artery bypass grafting (CABG) outcomes from the UK, with most studies originating from the USA, which is a predominantly private healthcare system. Consequently, the findings may not be directly applicable to the UK or European context, where universal public healthcare systems are more common.

The index of multiple deprivation (IMD) is the official measure used by the UK government to assess relative levels of deprivation across the general population residing in small areas of the nation, which are termed low-layer super output areas (LSOA). Each LSOA is scored, with an average population of 1500, ranked based on its weighted summary of 7 key domains. This seven distinct domains includes income deprivation (22.5%), employment deprivation (22.5%), education, skills and training deprivation (13.5%), health deprivation (13.5%), crime (9.3%), barriers to housing (9.3%) and services and living environment deprivation (9.3%). The IMD ranks every small area from 1, indicating the most deprived area, to 32 844, denoting the least deprived area. This is further divided into 10 equal deciles, with decile 1 representing the 10% most deprived area and decile 10 denoting the 10% least deprived area. Deprivation deciles are then further subdivided into five IMD-based deprivation groups, with group 1 encompassing decile 1 and 2, while group 5 includes decile 9 or 10 [9].

Coronary artery disease is strongly associated with major modifiable risk factors such as poorly controlled diabetes, hypertension, smoking and hyperlipidemia. These risk factors are disproportionately prevalent among socioeconomically deprived populations. Utilizing the IMD, we sought to determine if socioeconomic status (SES) is associated with a higher prevalence of modifiable risk factors, poorer in-hospital survival and early clinical outcomes following CABG in the UK. If SES was identified as a significant factor associated to the clinical outcome of interest, this paper will embark on its secondary objectives by examining if the incorporation of SES to EuroScore II would enhance in-hospital mortality risk prediction after cardiac surgery.

## METHODS

All patients who underwent elective or urgent isolated CABG from April 2008 to April 2019 were extracted from the National Adult Cardiac Surgery Audit (NACSA) database. The NACSA database prospectively collects data on all major heart operations carried out on National Health Service patients in the UK since April 1996. The definitions of database variables used and a description of the database were previously reported [10]. Essentially, data were imputed by healthcare professionals (surgeons, nurses, anaesthetists and perfusionists) by individual hospitals and then submitted to the NACSA central database. Patients who underwent emergency or salvage CABG, non-isolated CABG and had previous cardiac surgery were excluded to avoid introducing significant variability due to a small subset of the population and distinctive risk factors that do not represent the broader population who had undergone a planned procedure and allow a more homogenous cohort.

The seven domains in the IMD were divided into deciles, with 1 being the most deprived and 5 representing the least. The trend, hospital mortality and short-term clinical outcomes were evaluated against EuroScore II. SES, alongside EuroScore II variables, was input into a logistic regression model to assess the association between in-hospital mortality and socioeconomic deprivation.

SES, alongside EuroScore II variables, was input into logistic regression models to evaluate if it improves model performance. We compared the predictive performance of several models for in-hospital survival. The baseline model (Model 1) used the EuroScore II value. Subsequent models incorporated additional variables: Model 2 included all EuroScore II variables, and Model 3 included Model 2 with the addition of individual IMD components (income, employment, education, health, crime, housing and living environment).

### Ethical statement

The study was part of a research project approved by the Health Research Authority and Health and Care Research Wales. As the study included retrospective interrogation of the NICOR database, the need for individual patient consent was waived off (Health and Care Research Wales) (IRAS ID: 278171) in accordance with the research guidance. The study was performed in accordance with the ethical standards as laid down in the 1964 Declaration of Helsinki and its later amendments.

### Statistical analysis

Continuous variables are reported as mean and SD or median and IQR, pending the normality of the data. Categorical variables are reported as frequencies and percentages. The normality of the distribution of continuous data was assessed using the Shapiro–Wilk test.

The parametric analysis of variance (ANOVA) test was used to compare two or more independent normally distributed samples with the Kruskal–Wallis test used as a non-parametric counterpart. Tukey’s Honest Significant Difference test was further performed to control the family-wise error rate when multiple comparisons were performed to identify sub-groups with significant differences. The chi-square test of independence was used for categorical data with pairwise comparisons of proportions using the pairwise proportion test if the results were significant. Bonferroni correction was then applied after multiple pairwise comparisons. Multivariable logistic regression modelling was performed to examine the association between socioeconomic deprivation and in-hospital mortality among patients undergoing CABG. The model was adjusted for other key covariates included in the EuroScore II risk model, such as age, gender, renal function, diabetes and the New York Heart Association (NYHA) class (Table 1) as well as the individual seven components of the IMD. Results are demonstrated as odds ratio (OR) and 95% confidence interval (CI).

**Table 1:** The intra- and postoperative characteristics of patients who underwent isolated CABG according to the IMD-based deprivation groups

	Overall	1	2	3	4	5	*P*
On pump	149 840 (85.00%)	25 708 (86.72%)	25 752 (86.73%)	48 372 (84.37%)	25 877 (84.53%)	24 131 (83.24%)	<0.001
XClamp time (mins) (median, IQR)	47.00 (33.00, 62.00)	47.00 (33.00, 62.00)	45.00 (32.00, 60.00)	49.00 (36.00, 65.00)	45.00 (32.00, 60.00)	45.00 (31.00, 61.00)	<0.001
Bypass time (mins) (median, IQR)	80.00 (60.00, 101.00)	80.00 (60.00, 101.00)	79.00 (58.00, 99.00)	84.00 (63.00 , 105.00)	78.00 (58.00, 99.00)	78.00 (57.00, 100.00)	<0.001
Number of grafts (median, IQR)	3.00 (2.00 , 3.00)	3.00 (2.00 , 3.00)	3.00 (2.00 , 3.00)	3.00 (2.00 , 4.00)	3.00 (2.00 , 3.00)	3.00 (2.00 , 3.00)	<0.001
TLOS	8.00 (6.00 , 13.00)	9.00 (7.00 , 14.00)	8.00 (6.00 , 13.00)	8.00 (7.00 , 13.00)	8.00 (6.00 , 12.00)	8.00 (6.00 , 12.00)	<0.001
Multiple arterial grafts	22 793 (12.46%)	3059 (10.01%)	3283 (10.65%)	8967 (15.16%)	3742 (11.73%)	3742 (12.28%)	<0.001
In-hospital mortality	2246 (1.23%)	397 (1.30%)	401 (1.30%)	734 (1.24%)	362 (1.14%)	352 (1.15%)	<0.001
Return to theatre	6695 (3.91%)	1194 (4.15%)	1177 (4.13%)	2037 (3.73%)	1186 (3.93%)	1101 (3.80%)	0.008
CVA							0.072
TIA	640 (0.39%)	111 (0.39%)	102 (0.37%)	181 (0.34%)	128 (0.45%)	118 (0.43%)	
Stroke	863 (0.52%)	157 (0.56%)	154 (0.55%)	285 (0.53%)	152 (0.53%)	115 (0.42%)	
Postoperative dialysis	3182 (1.90%)	581 (2.04%)	578 (2.04%)	1067 (1.97%)	508 (1.74%)	448 (1.61%)	<0.001
Deep sternal wound infection	907 (1.02%)	154 (0.96%)	148 (1.01%)	317 (1.18%)	159 (1.00%)	129 (0.83%)	0.009

Group 1 denotes the most deprived, and 5 denotes the least deprived. XClamp: cross-clamp; TLOS: total length of stay; CVA: cerebrovascular accident; TIA: transient ischaemic attack.

To assess whether adding SES improves the performance of the logistic regression model, we plotted a receiver operating characteristic (ROC) curve for each model and compared prediction performance using the area under the curve (AUC). CIs for the AUC were calculated using the DeLong method. R (Version 4.2.3, R Foundation for Statistical Computing, Vienna, Austria) and R Studio (Version 1.4.1103, RStudio, PBC) were used for statistical analysis. Graphs and tables were created using R Studio (Version 1.4.1103, RStudio, PBC) and Microsoft Office 365 (Version 16.0.14026). A *P*-value of <0.05 is deemed statistically significant.

## RESULTS

**Figure 1:**
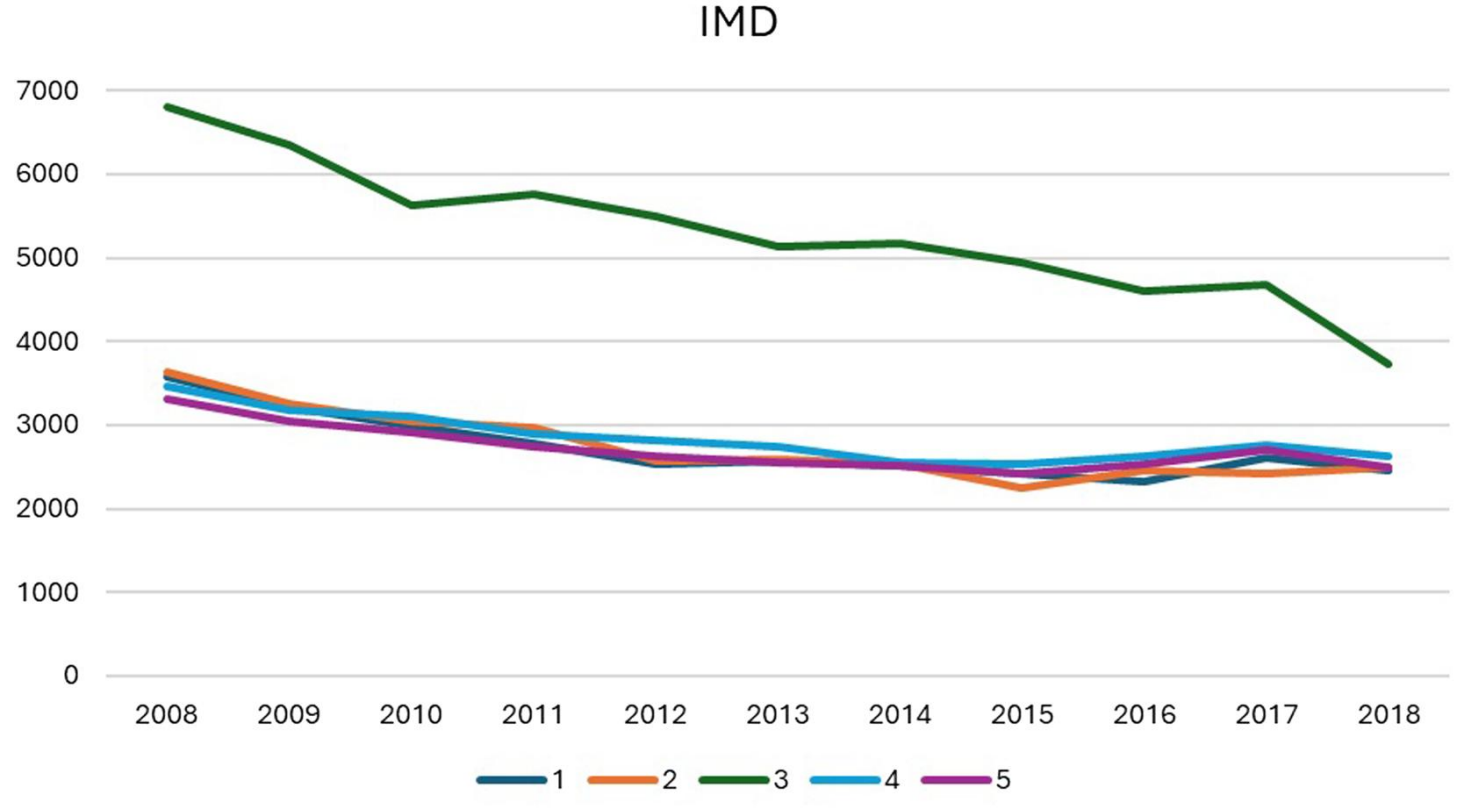
The number of patients in the 5 IMD-based deprivation groups index (1, 2, 3, 4, 5) of multiple deprivation groups who underwent isolated coronary artery bypass grafting in the UK from 2008–18.

A total of 182 911 patients (median age 67.3, 82.13% male) were included in the analysis, with 30 564, 30 815, 59 161, 31 891 and 30 480 patients categorized into IMD-based deprivation groups 1, 2, 3, 4 and 5, respectively. The patients’ characteristics are reported in Supplementary Material, Table S1. The number of isolated CABG procedures decreased from 20 777 in 2008 to 13 806 in 2018, with a notable decline in the group 3, where cases fell by 41.18% from 6355 annually. Conversely, the annual number of patients in IMD-based deprivation groups 1, 2, 4 and 5 remained relatively stable, ranging between 2500 and 3500 (Fig. 1).

**Figure 2:**
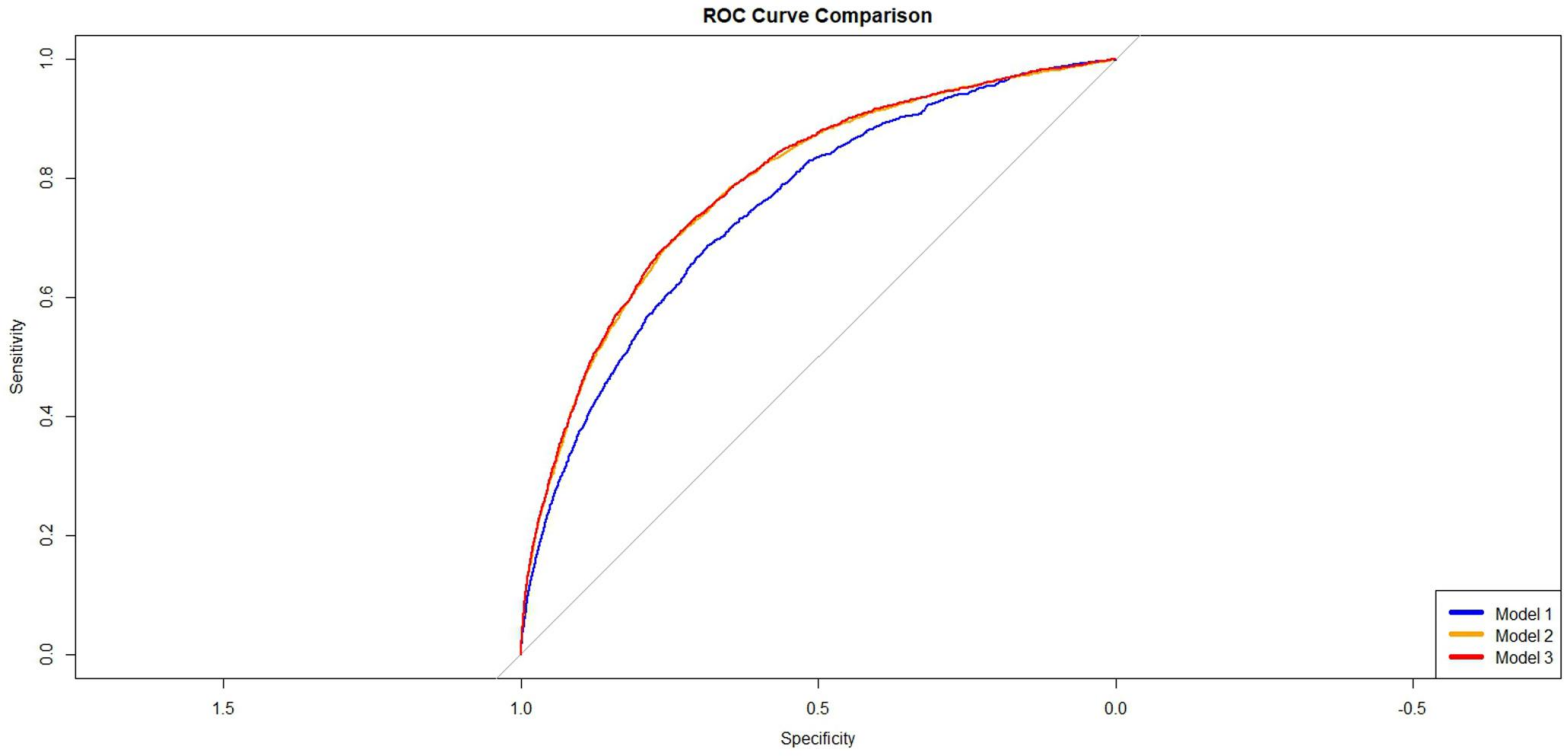
The receiver operating characteristics curve of the 3 models (Model 1: EuroScore II value, Model 2: EuroScore II characteristics, Model 3: Model 2 with individual 7 index of multiple deprivation components.).

The prevalence of urgent surgeries was higher in group 1, with 43.97% compared to 38.55% in group 5 (*P* < 0.001). Group 1 compared to group 5 also exhibited higher rates of chronic pulmonary disease requiring long-term medication (15.92% vs 9.03%), current smoking prevalence (20.93% vs 6.62%), diabetes management on insulin therapy (10.05% vs 6.10%) and median BMI higher (28.40 vs 27.94, *P* < 0.001). Additionally, 5.76% of patients in group 1 had a history of stroke, compared to 2.43% in group 5 and a history of myocardial infarction 11.82% vs 6.71% (both, *P* < 0.001). It is worth noting that the proportion of females who underwent surgical revascularization reduces as SES improves.

Overall in-hospital mortality was 1.23%, and the rates across IMD-based deprivation groups were 1.30% for group 1, 1.30% for group 2, 1.24% for group 3, 1.14% for group 4 and 1.15% for group 5 (*P* < 0.001). The in-hospital mortality rate decreased as SES improved. In addition, more cases were performed using an off-pump technique as the SES improved. Table 1 outlines the intra- and postoperative characteristics of patients in each IMD-based deprivation groups who underwent isolated CABG during the study period.

### Factors associated with in-hospital mortality

Several preoperative characteristics were associated with higher in-hospital mortality after isolated CABG. This includes (but not limited to) age (OR 1.07, 95% CI: 1.07–1.08, *P* < 0.001), female sex (OR 1.55, 95% CI: 1.40–1.70, *P* < 0.001), urgent operation (OR 1.41, 95% CI: 1.27–1.57, *P* < 0.001), diet control diabetes (OR 1.36, 95% CI: 1.12–1.62, *P* = 0.001) and preoperative atrial fibrillation (OR 1.57, 95% CI: 1.35–1.83, *P* < 0.001).

In terms of association between socioeconomic deprivation and in-hospital mortality, improvements in deprivation related to income were associated with a 20% reduction in odds of in-hospital mortality following isolated CABG in group 2 (OR 0.80, 95% CI: 0.64–0.99, *P* = 0.038), group 3 (OR 0.62, 95% CI: 0.47–0.82, *P* = 0.001), group 4 (OR 0.46, 95% CI: 0.33–0.64, *P* < 0.001) and group 5 (OR 0.48, 95% CI: 0.33–0.69, *P* < 0.001). Similarly, higher education levels were also linked to a 37% reduction in odds of in-hospital mortality in group 3 (OR 0.63, 95% CI: 0.34–0.88, *P* = 0.002), group 4 (OR 0.57, 95% CI: 0.21–0.85, *P* = 0.001) and group 5 (OR 0.44, 95% CI: 0.01–0.77, *P* < 0.001) (Table 2).

**Table 2:** The association of in-hospital mortality with patient characteristics and the index of multiple deprivations in seven domains

	Mortality		
Characteristics	Odds ratios	95% CI	*P*-value
Age	1.07	1.07–1.08	<0.001
Sex			
Male			
Female	1.55	1.40–1.70	<0.001
BMI	0.99	0.98–1.00	0.10
Left ventricular function			
Very poor (≤ 20%)			
Poor (21–30%)	0.43	0.22–0.93	0.02
Moderate (31–49%)	0.30	0.15–0.68	0.002
Good (>50%)	0.22	0.11–0.49	<0.001
Operative urgency			
Elective			
Urgent	1.41	1.27–1.57	<0.001
Diabetes			
Non-diabetic			
Diet control	1.36	1.12–1.62	0.001
Medication	1.09	0.97–1.22	0.13
Insulin use	1.75	1.53–1.99	<0.001
Smoking			
Non-smoker			
Ex-smoker	1.01	0.92–1.12	0.79
Current smoker	1.17	1.00–1.36	0.05
History of pulmonary disease	1.36	1.22–1.52	<0.001
Peripheral vascular disease	1.73	1.56–1.91	<0.001
Preoperative atrial fibrillation	1.57	1.35–1.83	<0.001
Neurological dysfunction preoperatively	0.84	0.49–1.50	0.53
CCS angina grade			
0			
1	0.78	0.64–0.96	0.02
2	0.74	0.64–0.86	<0.001
3	0.92	0.79–1.07	0.26
4	1.00	0.85–1.19	0.97
NYHA status			
1			
2	1.16	1.03–1.31	0.02
3	1.52	1.33–1.74	<0.001
4	2.28	1.88–2.77	<0.001
PCI			
No previous PCI			
PCI < 24 hours before surgery	1.59	0.84–2.72	0.12
PCI > 24 hours before surgery; same admission	1.02	0.72–1.40	0.90
PCI > 24 hours before surgery; previous admission	0.94	0.82–1.08	0.389
Previous MI			
None			
One	1.28	1.13–1.44	<0.001
Two or more	1.68	1.43–1.98	<0.001
Poor mobility	1.42	0.82–2.31	0.18
The interval between MI and surgery			
No previous MI			
MI < 6 hours	0.52	0.08–1.72	0.37
MI 6–24 hours	1.15	0.72–1.73	0.54
MI 1–30 days	0.85	0.74–0.97	0.02
MI 31–90 days	1.16	0.98–1.37	0.09
Ventilation preoperatively	1.19	1.03–1.65	0.03
Cardiogenic shock preoperatively	2.17	1.48–3.10	<0.001
Inotropes used preoperatively	2.74	1.95–3.76	<0.001
Income deprivation			
1			
2	0.8	0.64–0.99	0.04
3	0.62	0.47–0.82	0.001
4	0.46	0.33–0.64	<0.001
5	0.48	0.33–0.69	<0.001
Employment deprivation			
1			
2	0.96	0.77–1.20	0.71
3	1.09	0.82–1.46	0.56
4	1.14	0.82–1.60	0.43
5	1.14	0.78–1.68	0.50
Education, skills and training deprivation			
1			
2	0.99	0.85–1.21	0.88
3	0.63	0.34–0.88	0.002
4	0.57	0.21–0.85	0.001
5	0.44	0.01–0.77	<0.001
Health deprivation			
1			
2	1.15	0.96–1.38	0.13
3	1.17	0.94–1.45	0.15
4	1.11	0.87–1.41	0.40
5	1.1	0.84–1.45	0.48
Crime			
1			
2	0.95	0.81–1.11	0.51
3	0.92	0.79–1.09	0.34
4	1.1	0.93–1.31	0.25
5	0.93	0.77–1.12	0.44
Barriers to housing			
1			
2	1.02	0.88–1.19	0.78
3	0.94	0.81–1.10	0.44
4	0.92	0.78–1.07	0.29
5	0.92	0.78–1.07	0.29
Services and living environment deprivation			
1			
2	1.2	1.03–1.39	0.02
3	1.16	1.00–1.35	0.05
4	1.15	0.98–1.36	0.08
5	1.13	0.96–1.33	0.15

MI: myocardial infraction; NYHA: New York Heart Association; PCI: percutaneous coronary intervention; BMI: body mass index.

### Model performance with IMD

The AUC for models 1, 2 and 3 was 0.749 (95% CI: 0.739–0.759), 0.786 (95% CI: 0.777–0.796) and 0.789 (95% CI: 0.779–0.799), respectively. There was a small improvement in model performance between models 1,2, and 3. Figure 2 shows the ROC curve including all three models.

## DISCUSSION

Our results demonstrated that socioeconomic deprivation, particularly income and education deprivation, was associated with a small increase in in-hospital survival after elective/urgent, first-time, isolated CABG in the UK. The small percentage difference may look clinically irrelevant, but in the context of a large dataset, it equated to many more patients who did not survive the procedure (50 patients between IMD 1 and IMD 5).

Patients from the most socioeconomically deprived areas (Group 1) exhibited markedly higher rates of comorbidities and risk factors, including chronic pulmonary disease, active smoking, insulin-dependent diabetes, elevated BMI and a history of stroke. In addition, these patients also presented more frequently with catastrophic events like acute myocardial infarction and required urgent surgical intervention more frequently than their counterparts from less deprived areas. Notably, our study demonstrated that most patients receiving elective isolated CABG belonged to group 3, with fairly proportionate patient contribution from other IMD-based deprivation groups. Studies have shown that while the incidence and prevalence of cardiovascular disease are higher in greater deprivation groups, access to elective cardiac surgery is associated with less deprived groups. Our study also found that incorporating socioeconomic factors into the predictive model further improved its performance in predicting in-hospital mortality following CABG.

### The IMD

The IMD has gained widespread acceptance and is frequently employed to report socioeconomic deprivation across various surgical specialities in the UK. Its comprehensive nature makes it a valuable tool for understanding the multifaceted aspects of deprivation that may impact health outcomes [11, 12].

A similar index, known as the distressed communities index (DCI), has been increasingly used in evaluating economic disparities in the USA. This index consisted of seven components, including no high school diploma, housing vacancy rate, adults not working, poverty rate, median income ratio, changes in employment and changes in establishments. Cities and states were then classified into five tiers of well-being. The DCI has been demonstrated to be an effective tool in predicting survival and clinical outcomes after cardiac surgical and transcatheter procedures [2, 13, 14]. However, the DCI differs from the IMD in its focus and composition. While the DCI primarily emphasizes economic well-being, it does not explicitly incorporate health and living environment factors into its assessment as in the IMD.

### Socioeconomic deprivation in patients who underwent isolated CABG

SES comprises a range of factors that can vary significantly across different regions and cultural contexts [15, 16]. In developed countries, four key factors: income, education, employment status and environmental conditions have been consistently shown to be associated with cardiovascular diseases [8]. Our findings align with this trend, indicating that deprivation in both income and education is associated with poorer in-hospital outcomes following surgical revascularization in the UK. Areas with lower-income residents are significant predictors of 30-day mortality following CABG. Additionally, patients hailing from more deprived backgrounds often experience higher burdens of comorbidity and a greater likelihood of undergoing urgent surgeries.

Utilizing data from the US Nationwide Readmissions Database, a study has revealed that lower-income residential areas are significant predictors of 30-day mortality after CABG. Furthermore, similar to our study, patients hailing from more deprived backgrounds often experience higher burdens of comorbidity and a greater likelihood of undergoing urgent surgeries. Socioeconomic deprivation has also been associated with diminished preoperative quality of life [17], increased postoperative length of stay [6] and a reduction in access to cardiac surgery during the COVID-19 pandemic [2].

Although much of the existing (and the above) literature originates from the USA, where healthcare is largely private, the presence of publicly funded healthcare systems in Europe does not negate the existence of socioeconomic disadvantages. For example, a study conducted in 2000 in Italy found that the most socioeconomically disadvantaged groups faced higher 30-day mortality rates compared to those from more affluent backgrounds. This study underscored that universal health coverage does not automatically ensure equitable access to CABG [18]. Similarly, reports from the late 2000s in the UK revealed that lower SES was linked to poorer early outcomes following isolated CABG, as demonstrated by a single-centre study in northeastern Scotland [19]. Lai *et al.* found that female patients, black individuals and those from the most deprived areas had lower access to CABG and higher postoperative mortality [20]. Benedetto *et al.* further illustrated that payer status impacts hospital outcomes, highlighting persistent disparities even in countries with publicly funded healthcare systems, such as the UK [21]. Lastly, Dalen and colleagues have demonstrated a strong inverse association between income and mortality following cardiac surgery in Sweden [22].

While our report mainly addresses in-hospital outcomes, SES also affects long-term survival following isolated CABG. Pagano *et al.* tracked 44 902 adults who underwent cardiac surgery from 1997 to 2007 and identified social deprivation as a significant independent predictor of increased long-term mortality risk [23]. Similarly, Barnard *et al.* found that social deprivation correlates with reduced mid-term survival [24]. Long-term disparities may reflect ongoing challenges in achieving equity in healthcare delivery, despite advances in medical and surgical care. Future research should continue to explore these long-term outcomes and the underlying factors contributing to socioeconomic disparities in patients undergoing surgical revascularization.

### Limitations

Our study has several limitations. First and foremost, the use of the IMD to assess relative deprivation is designed to measure disparities at the area level, which may not accurately reflect individual circumstances. As a result, conclusions drawn at the neighbourhood level may not fully translate to the experiences of individuals within those areas. Second, the IMD is updated periodically rather than continuously. As a result, it may not capture recent changes in deprivation levels, potentially leading to a lag between actual socioeconomic conditions and those represented in our data. However, the highest proportions of neighbourhoods among the most deprived in England have remained largely consistent between the previous and the current IMD results [25] and largely mitigates concerns about the temporal limitations. Our findings are based on the UK healthcare system only, and this limits the generalizability of our results to other healthcare systems or countries with different measures of SES. Results may not be the same using our same methodology in other healthcare datasets. Lastly, accessibility to health care, including readmission after surgery as well as primary care utilization and long-term outcomes such as repeat revascularization, stroke and long-term survival, are not available from the national dataset we used, which limits the ability to assess the full impact of socioeconomic deprivation and other variables on patient outcomes.

## CONCLUSION

Socioeconomic deprivation, particularly related to income and education, is associated with a small decrease in in-hospital survival outcomes following isolated CABG. Furthermore, patients from more deprived backgrounds experience higher burdens of comorbidity and a greater likelihood of requiring urgent surgery. These findings underscore the critical need to address socioeconomic disparities in healthcare to improve outcomes for all patients. Ensuring equitable access to care and resources, regardless of SES, is essential for enhancing the effectiveness of surgical interventions.

## Supplementary Material

ivaf119_Supplementary_Data

## Data Availability

The data underlying this article were provided by the National Institute for Cardiovascular Outcomes Research under licence/by permission. Data will be shared on request to the corresponding author with the permission of the National Institute for Cardiovascular Outcomes Research.

## ABBREVIATIONS

AUC: Area under the curve
CABG: Coronary artery bypass grafting
CI: Confidence interval
DCI: Distressed communities index
IMD: Index of multiple deprivation
NACSA: National Adult Cardiac Surgery Audit
OR: Odds ratio
SES: Socioeconomic status
