# Supplementary material for: Socioeconomic deprivation is associated with worse in-hospital survival after isolated coronary artery bypass grafting in the UK
Source: Interdiscip Cardiovasc Thorac Surg. 2025 May 21;40(6):ivaf119. doi: 10.1093/icvts/ivaf119 (PMC12202203; doi:10.1093/icvts/ivaf119)
Supplement: ivaf119_Supplementary_Data [file ivaf119_supplementary_data.docx]

|  | Overall | 1 | 2 | 3 | 4 | 5 | p-value |
| --- | --- | --- | --- | --- | --- | --- | --- |
| Number of patients | 182911 | 30564 | 30815 | 59161 | 31891 | 30480 |  |
| Age (years) (Median, IQR) | 67.30 (60.00 , 74.00) | 64.40 (56.90 , 71.90) | 66.40 (58.90 , 73.40) | 67.40 (60.30 , 73.90) | 68.50 (61.50 , 74.80) | 69.30 (62.30 , 75.40) | <0.001 |
| Gender | | | | | | | <0.001 |
| Male | 150216 (82.13%) | 24252 (79.35%) | 25119 (81.52%) | 48639 (82.21%) | 26613 (83.45%) | 25593 (83.97%) |  |
| Female | 32695 (17.87%) | 6312 (20.65%) | 5696 (18.48%) | 10522 (17.79%) | 5278 (16.55%) | 4887 (16.03%) |  |
| BMI | 28.28 (25.65, 31.25) | 28.40 (25.88 , 32.03) | 28.28 (25.69 , 31.51) | 28.28 (25.79 , 31.48) | 28.28 (25.53 , 30.86) | 27.94 (25.32 , 30.47) | <0.001 |
| LVEFC | | | | | | | <0.001 |
| Very Poor (LVEF <21%) | 123 (0.07%) | 28 (0.09%) | 26 (0.08%) | 29 (0.05%) | 16 (0.05%) | 24 (0.08%) |  |
| Poor (LVEF 21 - 30%) | 44509 (24.33%) | 8222 (26.90%) | 7834 (25.42%) | 14707 (24.86%) | 7345 (23.03%) | 6401 (21.00%) |  |
| Moderate (LVEF 31 - 50%) | 5018 (2.74%) | 1101 (3.60%) | 880 (2.86%) | 1344 (2.27%) | 865 (2.71%) | 828 (2.72%) |  |
| Good (LVEF > 50%) | 133261 (72.86%) | 21213 (69.41%) | 22075 (71.64%) | 43081 (72.82%) | 23665 (74.21%) | 23227 (76.20%) |  |
| Urgency |  |  |  |  |  |  | <0.001 |
| Elective | 109491 (59.86%) | 17126 (56.03%) | 18062 (58.61%) | 36253 (61.28%) | 19320 (60.58%) | 18730 (61.45%) |  |
| Urgent | 73420 (40.14%) | 13438 (43.97%) | 12753 (41.39%) | 22908 (38.72%) | 12571 (39.42%) | 11750 (38.55%) |  |
| Diabetes |  |  |  |  |  |  | <0.001 |
| Not Diabetic | 129596 (70.85%) | 19916 (65.16%) | 20808 (67.53%) | 42187 (71.31%) | 23406 (73.39%) | 23279 (76.37%) |  |
| Diet Control | 7824 (4.28%) | 1314 (4.30%) | 1269 (4.12%) | 2725 (4.61%) | 1343 (4.21%) | 1173 (3.85%) |  |
| Oral therapy | 31030 (16.96%) | 6263 (20.49%) | 5902 (19.15%) | 9775 (16.52%) | 4920 (15.43%) | 4170 (13.68%) |  |
| Insulin therapy | 14461 (7.91%) | 3071 (10.05%) | 2836 (9.20%) | 4474 (7.56%) | 2222 (6.97%) | 1858 (6.10%) |  |
| Smoking |  |  |  |  |  |  | <0.001 |
| Never smoked | 63379 (34.65%) | 8301 (27.16%) | 9778 (31.73%) | 20838 (35.22%) | 11903 (37.32%) | 12559 (41.20%) |  |
| Ex smoker | 96722 (52.88%) | 15867 (51.91%) | 16636 (53.99%) | 31154 (52.66%) | 17162 (53.81%) | 15903 (52.18%) |  |
| Current smoker | 22810 (12.47%) | 6396 (20.93%) | 4401 (14.28%) | 7169 (12.12%) | 2826 (8.86%) | 2018 (6.62%) |  |
| Pulmonary Disease |  |  |  |  |  |  | <0.001 |
| No chronic pulmonary disease | 161432 (88.26%) | 25699 (84.08%) | 26810 (87.00%) | 52551 (88.83%) | 28643 (89.82%) | 27729 (90.97%) |  |
| Chronic pulmonary disease requiring use of long-term medication | 21479 (11.74%) | 4865 (15.92%) | 4005 (13.00%) | 6610 (11.17%) | 3248 (10.18%) | 2751 (9.03%) |  |
| PVD |  |  |  |  |  |  | <0.001 |
| No | 160350 (87.67%) | 25988 (85.03%) | 26913 (87.34%) | 51665 (87.33%) | 28365 (88.94%) | 27419 (89.96%) |  |
| Yes | 22561 (12.33%) | 4576 (14.97%) | 3902 (12.66%) | 7496 (12.67%) | 3526 (11.06%) | 3061 (10.04%) |  |
| Preop AF |  |  |  |  |  |  | <0.001 |
| No | 176531 (96.51%) | 29570 (96.75%) | 29802 (96.71%) | 57092 (96.50%) | 30658 (96.13%) | 29409 (96.49%) |  |
| Yes | 6380 (3.49%) | 994 (3.25%) | 1013 (3.29%) | 2069 (3.50%) | 1233 (3.87%) | 1071 (3.51%) |  |
| Neuro Dys |  |  |  |  |  |  | <0.001 |
| No | 178651 (97.67%) | 29728 (97.26%) | 30034 (97.47%) | 57845 (97.78%) | 31187 (97.79%) | 29857 (97.96%) |  |
| Yes | 4260 (2.33%) | 836 (2.74%) | 781 (2.53%) | 1316 (2.22%) | 704 (2.21%) | 623 (2.04%) |  |
| CCS Class |  |  |  |  |  |  | <0.001 |
| 0 | 18877 (10.32%) | 2701 (8.84%) | 2885 (9.36%) | 6471 (10.94%) | 3496 (10.96%) | 3324 (10.91%) |  |
| 1 | 16727 (9.14%) | 2514 (8.23%) | 2696 (8.75%) | 5441 (9.20%) | 3069 (9.62%) | 3007 (9.87%) |  |
| 2 | 70292 (38.43%) | 10532 (34.46%) | 11887 (38.58%) | 23041 (38.95%) | 12530 (39.29%) | 12302 (40.36%) |  |
| 3 | 54676 (29.89%) | 9966 (32.61%) | 9492 (30.80%) | 17100 (28.90%) | 9320 (29.22%) | 8798 (28.86%) |  |
| 4 | 22339 (12.21%) | 4851 (15.87%) | 3855 (12.51%) | 7108 (12.01%) | 3476 (10.90%) | 3049 (10.00%) |  |
| NYHA Class |  |  |  |  |  |  | <0.001 |
| 1 | 52960 (28.95%) | 8064 (26.38%) | 8702 (28.24%) | 16676 (28.19%) | 9771 (30.64%) | 9747 (31.98%) |  |
| 2 | 89455 (48.91%) | 14426 (47.20%) | 15058 (48.87%) | 29081 (49.16%) | 15751 (49.39%) | 15139 (49.67%) |  |
| 3 | 36020 (19.69%) | 7154 (23.41%) | 6257 (20.31%) | 11878 (20.08%) | 5744 (18.01%) | 4987 (16.36%) |  |
| 4 | 4476 (2.45%) | 920 (3.01%) | 798 (2.59%) | 1526 (2.58%) | 625 (1.96%) | 607 (1.99%) |  |
| PCI |  |  |  |  |  |  | <0.001 |
| No previous PCI | 156800 (85.72%) | 25690 (84.05%) | 26164 (84.91%) | 51242 (86.61%) | 27454 (86.09%) | 26250 (86.12%) |  |
| PCI < 24 hours before surgery | 623 (0.34%) | 97 (0.32%) | 104 (0.34%) | 162 (0.27%) | 121 (0.38%) | 139 (0.46%) |  |
| PCI > 24 hours before surgery; same admission | 2215 (1.21%) | 419 (1.37%) | 366 (1.19%) | 663 (1.12%) | 418 (1.31%) | 349 (1.15%) |  |
| PCI > 24 hours before surgery; previous admission | 23273 (12.72%) | 4358 (14.26%) | 4181 (13.57%) | 7094 (11.99%) | 3898 (12.22%) | 3742 (12.28%) |  |
| Previous MI |  |  |  |  |  |  | <0.001 |
| None | 91603 (50.08%) | 13113 (42.90%) | 14772 (47.94%) | 30147 (50.96%) | 16719 (52.43%) | 16852 (55.29%) |  |
| One | 77774 (42.52%) | 14344 (46.93%) | 13545 (43.96%) | 24675 (41.71%) | 13208 (41.42%) | 12002 (39.38%) |  |
| Two or more | 13534 (7.40%) | 3107 (10.17%) | 2498 (8.11%) | 4339 (7.33%) | 1964 (6.16%) | 1626 (5.33%) |  |
| Poor Mobility |  |  |  |  |  |  | <0.001 |
| No | 178117 (97.38%) | 29605 (96.86%) | 29922 (97.10%) | 57690 (97.51%) | 31102 (97.53%) | 29798 (97.76%) |  |
| Yes | 4794 (2.62%) | 959 (3.14%) | 893 (2.90%) | 1471 (2.49%) | 789 (2.47%) | 682 (2.24%) |  |
| Interval MI |  |  |  |  |  |  | <0.001 |
| No previous MI | 126871 (69.36%) | 19830 (64.88%) | 21078 (68.40%) | 41279 (69.77%) | 22586 (70.82%) | 22098 (72.50%) |  |
| MI < 6 hours | 123 (0.07%) | 25 (0.08%) | 25 (0.08%) | 36 (0.06%) | 21 (0.07%) | 16 (0.05%) |  |
| MI 6-24 hours | 828 (0.45%) | 160 (0.52%) | 154 (0.50%) | 269 (0.45%) | 140 (0.44%) | 105 (0.34%) |  |
| MI 1-30 days | 44180 (24.15%) | 8482 (27.75%) | 7745 (25.13%) | 13769 (23.27%) | 7465 (23.41%) | 6719 (22.04%) |  |
| MI 31-90 days | 10909 (5.96%) | 2067 (6.76%) | 1813 (5.88%) | 3808 (6.44%) | 1679 (5.26%) | 1542 (5.06%) |  |
| Ventilated reop |  |  |  |  |  |  | 0.459 |
| No | 182679 (99.87%) | 30527 (99.88%) | 30780 (99.89%) | 59087 (99.87%) | 31840 (99.84%) | 30445 (99.89%) |  |
| Yes | 232 (0.13%) | 37 (0.12%) | 35 (0.11%) | 74 (0.13%) | 51 (0.16%) | 35 (0.11%) |  |
| Cardiogenic Shock |  |  |  |  |  |  | 0.559 |
| No | 182316 (99.67%) | 30466 (99.68%) | 30718 (99.69%) | 58953 (99.65%) | 31800 (99.71%) | 30379 (99.67%) |  |
| Yes | 595 (0.33%) | 98 (0.32%) | 97 (0.31%) | 208 (0.35%) | 91 (0.29%) | 101 (0.33%) |  |
| Inotropes |  |  |  |  |  |  | 0.39 |
| No | 182203 (99.61%) | 30434 (99.57%) | 30708 (99.65%) | 58925 (99.60%) | 31762 (99.60%) | 30374 (99.65%) |  |
| Yes | 708 (0.39%) | 130 (0.43%) | 107 (0.35%) | 236 (0.40%) | 129 (0.40%) | 106 (0.35%) |  |
| Income deprivation |  |  |  |  |  |  | <0.001 |
| 1 (Most deprived) | 30300 (16.57%) | 27331 (89.42%) | 2969 (9.63%) | 0 (0.00%) | 0 (0.00%) | 0 (0.00%) |  |
| 2 | 31037 (16.97%) | 3184 (10.42%) | 22371 (72.60%) | 5440 (9.20%) | 42 (0.13%) | 0 (0.00%) |  |
| 3 | 59373 (32.46%) | 48 (0.16%) | 5036 (16.34%) | 46763 (79.04%) | 7346 (23.03%) | 180 (0.59%) |  |
| 4 | 32216 (17.61%) | 1 (0.00%) | 420 (1.36%) | 5917 (10.00%) | 18404 (57.71%) | 7474 (24.52%) |  |
| 5 (Least deprived) | 29985 (16.39%) | 0 (0.00%) | 19 (0.06%) | 1041 (1.76%) | 6099 (19.12%) | 22826 (74.89%) |  |
| Employment deprivation |  |  |  |  |  |  | <0.001 |
| 1 (Most deprived) | 31692 (17.33%) | 27007 (88.36%) | 4652 (15.10%) | 33 (0.06%) | 0 (0.00%) | 0 (0.00%) |  |
| 2 | 31512 (17.23%) | 3484 (11.40%) | 19883 (64.52%) | 7768 (13.13%) | 377 (1.18%) | 0 (0.00%) |  |
| 3 | 59581 (32.57%) | 65 (0.21%) | 5473 (17.76%) | 43727 (73.91%) | 9755 (30.59%) | 561 (1.84%) |  |
| 4 | 31839 (17.41%) | 8 (0.03%) | 745 (2.42%) | 6028 (10.19%) | 15538 (48.72%) | 9520 (31.23%) |  |
| 5 (Least deprived) | 28287 (15.46%) | 0 (0.00%) | 62 (0.20%) | 1605 (2.71%) | 6221 (19.51%) | 20399 (66.93%) |  |
| Education, skills and training deprivation | | | | | | | <0.001 |
| 1 (Most deprived) | 30722 (16.80%) | 23062 (75.45%) | 6719 (21.80%) | 937 (1.58%) | 4 (0.01%) | 0 (0.00%) |  |
| 2 | 32224 (17.62%) | 6019 (19.69%) | 14044 (45.58%) | 9531 (16.11%) | 2368 (7.43%) | 262 (0.86%) |  |
| 3 | 58506 (31.99%) | 1220 (3.99%) | 6964 (22.60%) | 38302 (64.74%) | 9286 (29.12%) | 2734 (8.97%) |  |
| 4 | 31846 (17.41%) | 226 (0.74%) | 2541 (8.25%) | 7683 (12.99%) | 12305 (38.58%) | 9091 (29.83%) |  |
| 5 (Least deprived) | 29613 (16.19%) | 37 (0.12%) | 547 (1.78%) | 2708 (4.58%) | 7928 (24.86%) | 18393 (60.34%) |  |
| Health deprivation |  |  |  |  |  |  | <0.001 |
| 1 (Most deprived) | 32028 (17.51%) | 24399 (79.83%) | 6884 (22.34%) | 700 (1.18%) | 45 (0.14%) | 0 (0.00%) |  |
| 2 | 30899 (16.89%) | 5257 (17.20%) | 13909 (45.14%) | 8627 (14.58%) | 2847 (8.93%) | 259 (0.85%) |  |
| 3 | 58621 (32.05%) | 832 (2.72%) | 7171 (23.27%) | 38038 (64.30%) | 9465 (29.68%) | 3115 (10.22%) |  |
| 4 | 31072 (16.99%) | 74 (0.24%) | 2584 (8.39%) | 8240 (13.93%) | 11308 (35.46%) | 8866 (29.09%) |  |
| 5 (Least deprived) | 30291 (16.56%) | 2 (0.01%) | 267 (0.87%) | 3556 (6.01%) | 8226 (25.79%) | 18240 (59.84%) |  |
| Crime |  |  |  |  |  |  | <0.001 |
| 1 (Most deprived) | 29194 (15.96%) | 18398 (60.20%) | 7341 (23.82%) | 2497 (4.22%) | 892 (2.80%) | 66 (0.22%) |  |
| 2 | 29735 (16.26%) | 7992 (26.15%) | 10267 (33.32%) | 7029 (11.88%) | 3636 (11.40%) | 811 (2.66%) |  |
| 3 | 58258 (31.85%) | 3030 (9.91%) | 7269 (23.59%) | 36102 (61.02%) | 7760 (24.33%) | 4097 (13.44%) |  |
| 4 | 32009 (17.50%) | 999 (3.27%) | 3594 (11.66%) | 7617 (12.88%) | 10113 (31.71%) | 9686 (31.78%) |  |
| 5 (Least deprived) | 33715 (18.43%) | 145 (0.47%) | 2344 (7.61%) | 5916 (10.00%) | 9490 (29.76%) | 15820 (51.90%) |  |
| Barriers to housing |  |  |  |  |  |  | <0.001 |
| 1 (Most deprived) | 30581 (16.72%) | 5610 (18.35%) | 8463 (27.46%) | 8614 (14.56%) | 6041 (18.94%) | 1853 (6.08%) |  |
| 2 | 29420 (16.08%) | 5665 (18.53%) | 5193 (16.85%) | 6246 (10.56%) | 6779 (21.26%) | 5537 (18.17%) |  |
| 3 | 57257 (31.30%) | 6833 (22.36%) | 5187 (16.83%) | 32324 (54.64%) | 6060 (19.00%) | 6853 (22.48%) |  |
| 4 | 31756 (17.36%) | 6938 (22.70%) | 5481 (17.79%) | 5644 (9.54%) | 6077 (19.06%) | 7616 (24.99%) |  |
| 5 (Least deprived) | 33897 (18.53%) | 5518 (18.05%) | 6491 (21.06%) | 6333 (10.70%) | 6934 (21.74%) | 8621 (28.28%) |  |
| Services and living environment deprivation | | | | | | | <0.001 |
| 1 (Most deprived) | 29439 (16.09%) | 9849 (32.22%) | 8543 (27.72%) | 7706 (13.03%) | 3103 (9.73%) | 238 (0.78%) |  |
| 2 | 30402 (16.62%) | 6524 (21.35%) | 7983 (25.91%) | 7572 (12.80%) | 6345 (19.90%) | 1978 (6.49%) |  |
| 3 | 59167 (32.35%) | 5660 (18.52%) | 5946 (19.30%) | 34045 (57.55%) | 7964 (24.97%) | 5552 (18.22%) |  |
| 4 | 31947 (17.47%) | 4360 (14.27%) | 4546 (14.75%) | 5791 (9.79%) | 7703 (24.15%) | 9547 (31.32%) |  |
| 5 (Least deprived) | 31956 (17.47%) | 4171 (13.65%) | 3797 (12.32%) | 4047 (6.84%) | 6776 (21.25%) | 13165 (43.19%) |  |

Supp Table 1 shows the pre-operative characteristics of patients who underwent isolated CABG according to the quintile index (1,2,3,4,5) of multiple deprivation.
